# Prevalence of Cognitive Frailty, Do Psychosocial-Related Factors Matter?

**DOI:** 10.3390/brainsci10120968

**Published:** 2020-12-11

**Authors:** Esperanza Navarro-Pardo, David Facal, María Campos-Magdaleno, Arturo X. Pereiro, Onésimo Juncos-Rabadán

**Affiliations:** 1 Department of Developmental and Educational Psychology, Universitat de Valencia, 46010 Valencia, Spain; Esperanza.Navarro@uv.es; 2 Department of Developmental Psychology, University of Santiago de Compostela, 15782 Santiago de Compostela, Spain; maria.campos@usc.es (M.C.-M.); arturoxose.pereiro@usc.es (A.X.P.); onesimo.juncos@usc.es (O.J.-R.)

**Keywords:** CogniFraSp, cognitive frailty, older adults, psychosocial factors, prevalence

## Abstract

Cognitive frailty (CF) is a topic of growing interest with implications for the study of preventive interventions in aging. Nevertheless, little research has been done to assess the influence of psychosocial variables on the risk of CF. Our objectives were to estimate the prevalence of CF in a Spanish sample and to explore the influence of psychosocial variables in this prevalence. Physical frailty and cognitive, functional, psychosocial, and socio-demographic aspects were assessed in a sample of 285 participants over 60 years. Univariate and multivariate logistic regression models were carried out. A prevalence of 21.8% (95% CI 17.4–26.9) was established when both frail and pre-frail conditions were included, and a prevalence of 3.2% (95% CI 1.7–5.9) if only frail individuals were considered. Age, educational level, profession and psychological well-being variables significantly predicted CF. Frailty and pre-frailty are high-prevalence health conditions in older adults influenced by socio-demographic, socio-educative and affective factors.

## 1. Introduction

Research perspectives on frailty are making relevant contributions to understand the major domains that mediate the complex relationships between aging, on the one hand, and physical and psychological conditions, on the other one. Frailty is conventionally defined as a multidimensional clinical syndrome, characterized by loss of biophysical reserve and diminished resistance to stressors, causing vulnerability to adverse health outcomes and leading to loss of function that can be expressed in different ways (i.e., energy, physical ability, cognition, and health) [1,2]. Frailty itself can be classified as physical frailty, cognitive frailty, or psychosocial frailty [3]. Therefore cognitive frailty (CF) has been considered as a subtype of frailty [4] and it would consist of a heterogeneous clinical condition characterized by the simultaneous presence of both physical frailty and mild cognitive impairment (MCI) (CDR = 0.5), once the diagnosis of dementia due to Alzheimer’s disease (AD) or other dementias and the condition of physical disability have been excluded [5].

The prevalence of CF was recently estimated to be 1% to 5% in community-dwelling older adults [6,7,8,9]. However, a relevant heterogeneity (ranging from 0.9% to 40.0%) is evident in the literature and several factors have probably contributed to such wide variability; thus, there have been differences in the way the two components of cognitive frailty have been operationalized [10], and some of them have also included physical pre-frailty [11,12]. Likewise, prevalence data should be affected by the settings differences. Some studies were carried out in clinical settings [11] while others were community-based studies [13,14]. In the last ones, including individuals with physical frailty and pre-frailty, the prevalence of CF ranged from 1 to 40% [14], whereas in clinical-based studies it ranged from 10.7 to 22.0% [13]. Almost all studies found that age was significantly associated with CF [15,16] and prevalence increased with age [8], but regarding to the sex and CF association the results are not yet conclusive [8,13].

CF can be influenced by several physical risk factors and by psychosocial factors [6], intensifying the vulnerability to stressors [7]. A key consideration when addressing CF is how intrinsic factors as age, sex, or medical and functional capabilities interact with extrinsic factors such as social support, education level, or occupational category. In order to get a holistic understanding of the relationship between aging and frailty, the complexity of social and psychological factors must be considered. Thus, considering that CF is characterized by reduced cognitive reserve [5], older people with lower educational level attainment and who carried out non-intellectual jobs [7,17] would tend to show increased CF risks.

There is also evidence of relationships between the role of the family and broader social networks and frailty in later life. In this context, social engagement emerges as a factor with a protective or balancing function in the CF levels [17]. However, these social networks can diminish in later life. In fact, increasing aging has been related with a decrease in several ways of social support [9] (e.g., loss of partner or other family members or friends), isolation [18], or living alone [19] that may lead to a decline in physical and mental functions.

Additionally, research on CF requires a more comprehensive assessment of psychological well-being in order to capture psychological aspects of cognitive vulnerability. According to this, some studies have pointed out the significant association between CF and mental health status, and specifically with depressive mood, anxiety, impatience, behavioral suppression and reduced desire to participate in social activities [20]. Although the influence of psychosocial variables on CF has appeared in cross-sectional [19,21] and cohort studies on frailty, as far as we know, no study has been designed to specifically study this relationship.

The construct of CF has triggered growing interest in the scientific community, but only a limited amount of evidence on its prevalence and the relationships between psychosocial conditions and CF is available. Furthermore, no previous study has analyzed the prevalence of CF in the Spanish context. The aims of this study were to establish the prevalence rate of CF on a sample of Spanish community-dwelling older adults and to gain knowledge about the role of psychosocial variables on CF.

## 2. Materials and Methods

### 2.1. Sample

The sample comprised 285 community-dwelling participants (from Galicia, NW of Spain and from Valencia, SE of Spain) aged 60 years or more, without diagnosis of dementia, or major mental health disorders. Other exclusion criteria were to have traumatic injuries, non-compensable sensory or motor deficiencies that prevent evaluation, serious gait disturbances (inability to walk more than 10 m without help, obvious risk of falling); to use technical assistance (cane, crutches, walker, wheelchair); and to suffer disability and/or recognized dependency for instrumental activities of daily life. Sampling was incidental and was recruited during 2018 and 2019. Community-dwelling participants were recruited from a large on-going study on cognitive aging being undertaken at the University of Santiago de Compostela and University of Valencia. Candidates were relatives or neighbors of university students and were invited to participate in the study when active life and the autonomy for the instrumental activities of daily life were maintained. The participants were evaluated in their own homes and received no incentives for their collaboration in the study.

### 2.2. Measures

A customized protocol was developed to measure aspects related with cognitive performance, psycho-social related factors and physical frailty. The Montreal Cognitive Assessment (MoCA) test was administered to determine possible MCI participants [10]. To measure psychological well-being, the Spanish version of the General Health Questionnaire (GHQ-12) was used [22]; the GHQ-12 [23] is a shorten version of the GHQ, a self-report questionnaire used to assess psychological well-being level including perceived stress, anxiety level, feelings of fear, sleep disturbances, or psychosomatic conditions. Social support was assessed with the Spanish version of the MOS questionnaire (Medical Outcomes Study Social Support Survey, MOS-SS) that is a five-point Likert scale and permits detection of situations characterized by elevated social risk [24]. According to the authors, a cut-off score of 57 points was used for lack of social support. Age and years of education were also recorded.

Because this study is part of a larger research, physical frailty was assessed following a modified version of the frailty phenotype as described by Fried et al. [1]; particularly physical endurance was measured with GHQ items and (not with GDS-Geriatric Depression Scale ones) and physical activity was measured with a specific questionnaire (Spanish Short Version of Minnesota Leisure Time Activities Questionnaire) and not only with a self-informed item. The following criteria were used: (1) Weight loss, measured with yes/no responses about unintentional weight loss and lack of appetite in the last three months; (2) Self-reported exhaustion, measured with a question about depressed affective state from the GHQ-12 [22]; (3) Weakness, measured by the grip strength of the dominant hand, 3 measurements are taken and the average is obtained; (4) Slow gait speed, measured through a timed-up and go (TUG) task in which the participant have to get up from a chair, walk three meters, turn on himself, step back, and sit back down [25]; and (5) Low physical activity, measured with the short Spanish version of the Minnesota leisure time Physical Activity Questionnaire (VREM) [26].

Regarding cognitive status, a reference value for possible cognitive impairment was established for MoCA scores below the 5th percentile (−1.64 standard deviations), adjusted for age and educational level according to the normative scores by Pereiro et al. [27]. Regarding frailty status, the involuntary weight loss criterion is considered to be met when the response to the question, “Have you eaten less due to poor appetite, digestive problems, chewing or swallowing difficulties in the last 3 months?” or to the question about recent weight loss is positive; the criterion for low mood is considered to be met when a negative answer is recorder to question 9 of the GHQ12, “Have you felt unhappy and depressed?”; the criterion of grip strength is considered to be met if the performance is below that expected according to the FNIH criteria (men < 26, women < 16) [28]; the criterion of low physical activity is considered to be met if the participant is classified through the VRM as Sedentary [27]; finally, the criterion of slow mobility is considered to be met when the performance in the TUG is below the expected value according to age and sex in a normative sample of more than 50 years, taking as a cut-off point the percentile 84 (1 standard deviation) adjusted for age and sex [29]. TUG performance is considered above the range, in men: 50 to 54 = 7; 55 to 59 = 8; 60 to 69 = 9; 70 to 79 = 10; 80 ± 11. According to the number of criteria that the participant meets, it will be considered: physically robust, if they do not meet any criteria; physically pre-frail, if you meet 1 or 2 criteria; physically frail, if you meet 3, 4, or 5 criteria.

### 2.3. Study Design and Procedure

A more extensive cross-sectional study (CogniFraSp, Cognitive Frailty Spain) was conducted during the years 2018 and 2019, assessing cognitive, functional, and psychosocial aspects using valid assessment tools and adapted self-reported tests. The study received approval by the Ethics in Clinical Research Committee of the Galician Government (2018/620) and the Ethics Committee of University of Valencia (H1521026499251), and was conducted in accordance with the Declaration of Helsinki. Written informed consent was obtained from all participants.

Following the five Fried criteria, participants were classified as non-frail, pre-frail, and frail when they met no criteria, one or two criteria and three, four or five criteria respectively. According to their non-frail, pre-frail or frail phenotype and their cognitive status, participants were classified as (a) Non-frail-cognitively unimpaired, (b) Pre-frail-cognitively unimpaired, (c) Frail-cognitively unimpaired, (d) Non-frail-possible cognitive impairment, (e) Pre-frail-possible cognitive impairment, and (f) Frail-possible cognitive impairment. According to the general criteria established by Kelaiditi et al. [5], we considered as cases of cognitive frailty those with a frail and pre-frail phenotype and possible cognitive impairment.

### 2.4. Study Outcomes

Age was categorized according to five age groups (60–64, 65–69, 70–74, 75–79, 80+ years old) and gender was dichotomized in men and women. Regarding formal education, considering the current levels of formal education in old adults in Spain [30], education was dichotomized in low (7 or less years of education) and high (more than 7 years of education). Profession was dichotomized in low qualification (including unskilled worker, housewife, and no employment) and high qualification (including skilled worker, trader, senior and middle-level civil servant, administrative, and management staff).

In relation to Charlson Comorbidity Index, its scores were used to categorize the sample in three groups (No chronic conditions for 0 scores, One chronic condition for score of 1, Two or more chronic conditions for scores higher than 1).

In terms of instrument measurements, social support status was dichotomized according to MOS-SS scores (With social support for scores of 57 or more, Without social support for scores of 56 or less), while psychological well-being status was dichotomized according to GHQ-12 scores, using the recommended cut-off threshold for mental health issues (2/3 points), with responses about the presence of different affective symptoms scored as 0, 0, 1, and 1 respectively [23].

### 2.5. Statistical Analysis

Statistical analyses were carried out with SPSS for Windows, version 26.0 (IBM corp., Armonk, NY, USA). The significance level was established at 0.05 for all analyses. The χ^2^ test was used for categorical variables. Prevalence was estimated from the number and percentage of cases and odds ratios (OR), with 95% confidence intervals. Logistic regression analyses were performed to predict the presence of CF. Participants with CF (pre-frail and frail phenotype with cognitive impairment) were considered cases and those participants without cognitive impairment (non-frail-cognitively unimpaired, pre-frail-cognitively unimpaired, and frail-cognitively unimpaired) no cases. Age, gender, years of formal education, profession, social support status, and psychological well-being level were entered as independent variables in univariate analyses. Then, multivariate logistic regression models were performed including only those independent variables considered predictors because of a significant relation (*p* < 0.05) in the previous univariate analyses and adjusting for possible confounding.

## 3. Results

Sociodemographic characteristics (age, gender, years of education, and profession) and levels of comorbidity according to the Charlson Comorbidity Index, physical status, social support, and psychological well-being are shown in Table 1. The prevalence of CF was 21.8% (95% CI 17.4–26.9), with 62 cases from a total sample of 285 participants. Of those, 9 cases (prevalence 3.2%, 95% CI 1.7–5.9) presented cognitive impairment and physical frailty and 53 cases (prevalence 18.6%, 95% CI 14.5–23.5) presented cognitive impairment and pre-physical frailty. The results concerning the roles of the independent variables in the prevalence of CF are shown in Table 2. Significant differences were found for age (χ^2^ = 19.83; *p* = 0.001), with the 80+ year groups showing a significantly higher CF prevalence; for level of formal education (χ^2^ = 24.22; *p* < 0.001), with low education presenting higher CF prevalence than high education; for profession (χ^2^ = 14.71; *p* < 0.001) with low qualification professions presenting a higher CF prevalence than high qualification professions; for psychological well-being (χ^2^ = 7.83; *p* = 0.005), with higher prevalence in those subgroup presenting low psychological well-being according to GHQ-12 scores. Gender and comorbidity differences were not significant.

According to the results from univariate models, only the significant independent variables, age group, formal education, profession, and psychological well-being were included in a multivariate logistic regression model (see Table 3). The final model shows a 4.24 increased risk in participants of 80 and more years compared with participants in the 60–64 age group, a 3.43 increased risk in participants with low formal education compared to participants with high education, a 2.56 increased risk in participants with low qualified professions compared with participants with high qualified works, and an increased risk of 2.94 in participants with low psychological well-being compared with participants with high psychological well-being. The model achieved a 76.6% of correct classifications of the participants using age, formal education, profession, and psychological well-being as predictors, with Hosmer–Lemeshow statistic indicating a good fit (χ^2^ = 8.79; *sig.* = 0.36).

## 4. Discussions

Our study explored CF prevalence on a Spanish sample of older adults, and predictive associations with psychosocial variables such as educational level, developed profession, social support, and psychological well-being status were analyzed.

Our prevalence results are in line with most published studies in community-dwelling settings [14,15]. Thus, higher CF prevalence is found when both frail and pre-frail conditions are included (21.8%), whereas much lower values are found when only frail individuals are considered (3.2%).

Regarding age, as expected [8,15,16], significant increased risk is found in the oldest when compared with the youngest group. Regarding gender, our study showed more favorable trends in men but, as in previous studies [8,19], no significant gender differences in CF risk were found.

As reported in previous studies [7,17], lower socio-cultural status, measured by low education level and low professional qualification, is associated with CF both in univariate and multivariate models. The link is probably not direct but could occur through mediating and moderating variables as lifestyle factors related to that status (e.g., access to health services and active aging activities, work conditions, and work complexity, encouraging opportunities of the environment, accessibility and security of the home environments, alcohol or other drugs use, patterns of nutrition, and levels of physical exercise). In light of the results, social factors might represent risk factors for the development of CF, and therefore social profile should be systematically assessed and taken into account when evaluating old adults for the development and implementation of multidimensional prevention and treatment programs [19]. Furthermore, low level of education and low professional attainment as two core proxies of reduced cognitive reserve, appeared as significant predictors of CF in both uni- and multivariate analyses [31,32,33].

Concerning psychological well-being levels, a 16.8% of our sample showed low psychological wellbeing, and this variable significantly predicted CF in both univariate and multivariate models. Furthermore, a 39.6% of people with low level of psychological wellbeing showed CF symptoms. In line with a broader view of frailty, mental health emerges as an important variable in CF [20,34], and the psychological status of individuals [9] should be included in CF assessments. Future studies should delve into the direction of the relationship.

In relation to health status, significant relationships were not found with physical comorbidity probably due to our objective was to investigate CF prevalence on community-dwelling population, almost all free of chronic health conditions.

Similarly, our results did not show significant association between social support and CF. However, it is interesting to note that the whole sample showed a very high rate of perceived social support (93.3%), which is probably related to cultural factors (high density of social contacts, family-oriented models of preference with high contacts with the closest members of the family of origin) and may positively influence the psychological and cognitive status of Spanish older people [35,36].

As to the limitations of the study, although the size sample is acceptable to the objectives, it would be desirable to extend the study with larger samples recruited by representative sampling procedures to gain greater awareness of CF prevalence and psychosocial factors related. On the other hand, as far as their relation to the prevalence found, the exclusion criteria used are coherent with the detection of fragility and cognitive fragility, as intended in this study but, consequently, would exclude a part of elderly people with some motor and/or mental health comorbidities.

## 5. Conclusions

A CF prevalence of 21.8% was established in a sample of Spanish community-dwelling old adults when both frail and pre-frail conditions were included. A CF prevalence of 3.2% was established when only frail individuals were considered. Age, educational level, profession and psychological well-being significantly predicted CF. The role of lower socio-cultural status, measured by low education level and low professional qualification, and low psychological well-being levels must be taken into account in CF assessment.

## Figures and Tables

**Table 1:** Demographic profile, cognitive and physical status, social support, and psychological well-being.

Demographic Characteristics	Frequencies (%)
Age groups	
60–64 years	56 (19.6%)
65–69 years	58 (20.4%)
70–74 years	57 (20.0%)
75–79 years	53 (18.6%)
80 + years	61 (21.4%)
Gender	
Men	132 (46.3%)
Women	153 (53.7%)
Formal education	
Low educ. level	137 (51.7%)
High educ. level	128 (48.3%)
Profession	
Low qualification	161 (56.5%)
High qualification	124 (43.5%)
Comorbidity	
No chronic conditions	180 (63.2%)
One chronic condition	74 (26.0%)
Two or more chronic conditions	31 (10.9%)
**Cognitive and frailty status**	
Non-frail-cognitively unimpaired	80 (28.1%)
Pre-frail-cognitively unimpaired	109 (38.2%)
Frail-cognitively unimpaired	14 (4.9%)
Non-frail-cognitive impairment	20 (7.0%)
Pre-frail-cognitive impairment	53 (18.6%)
Frail-possible cognitive impairment	9 (3.2%)
**Social support and psychological well-being**	
Social support status	
With social support	266 (93.3%)
Without social support	19 (6.7%)
Psychological well-being	
High well-being	237 (83.2%)
Low well-being	48 (16.8%)

**Table 2:** Prevalence of cognitive frailty (CF) according to age, gender, formal education, profession, social support status, and psychological well-being (univariate logistic regression).

Covariates	Cases	%	Wald’s	*p*-Values	OR	95% CI
Age group						
60–64	7/53	13.2			1	
65–69	7/54	13	0.01	0.97	0.98	0.32–3.01
70–74	14/52	26.9	2.98	0.084	2.42	0.89–6.61
75–79	9/49	18.4	0.51	0.476	1.48	0.51–4.33
80+	25/57	43.9	11.35	0.001	5.13	1.98–13.30
Gender						
Men	22/123	17.9			1	
Women	40/142	28.2	3.83	0.050	0.55	0.31–1.00
Formal education						
Low educ. level	49/137	35.8	21.66	0.001	4.93	2.52–9.64
High educ. level	13/128	10.2			1	
Profession						
Low qualification	47/146	32.2	13.18	<0.001	3.29	1.70–6.26
High qualification	15/119	12.6			1	
Comorbidity						
No conditions	38/161	23.6			1	
One condition	17/74	23.0	0.001	0.975	1.02	0.40–2.55
Two or more cond.	7/30	23.3	0.002	0.968	0.98	0.36–2.68
Social support						
With support	58/247	23.5		1		
Without support	4/18	22.2	0.015	0.903	1.07	0.34–3.39
Psychological well-being						
Low well-being	19/48	39.6	8.19	0.01	2.65	1.36–5.17
High well-being	43/217	19.8		1		

**Table 3:** Final multivariate logistic regression model.

Covariates	B	S.E.	Wald’s	*p*-Values	OR	95% CI
Age group						
60–64					1	
65–69	−0.20	0.61	0.11	0.74	0.99	0.31–3.15
70–74	0.56	0.55	1.04	0.31	2.10	0.74–5.96
75–79	−0.16	0.59	0.08	0.78	1.21	0.40–3.67
80+	1.00	0.53	6.28	0.05	4.24	1.57–11.44
Formal education						
Low	1.23	0.38	10.59	0.01	3.43	1.63–7.21
High					1	
Profession						
Low qualification	0.94	0.38	6.28	0.001	2.56	0.97–7.70
High qualification					1	
Psychol. well-being						
No problems					1	
Mental health problems	1.08	0.40	7.43	0.001	2.94	1.35–6.39
